# A mechanistic model of heat transfer for gas–liquid flow in vertical wellbore annuli

**DOI:** 10.1007/s12182-017-0193-y

**Published:** 2017-10-27

**Authors:** Bang-Tang Yin, Xiang-Fang Li, Gang Liu

**Affiliations:** 1 School of Petroleum Engineering, China University of Petroleum (East China), Qingdao, 266580 Shandong China; 2 College of Petroleum Engineering, China University of Petroleum, Beijing, 102249 China

**Keywords:** Gas–liquid flow, Vertical annuli, Heat transfer, Tubing liquid film, Casing liquid film

## Abstract

The most prominent aspect of multiphase flow is the variation in the physical distribution of the phases in the flow conduit known as the flow pattern. Several different flow patterns can exist under different flow conditions which have significant effects on liquid holdup, pressure gradient and heat transfer. Gas–liquid two-phase flow in an annulus can be found in a variety of practical situations. In high rate oil and gas production, it may be beneficial to flow fluids vertically through the annulus configuration between well tubing and casing. The flow patterns in annuli are different from pipe flow. There are both casing and tubing liquid films in slug flow and annular flow in the annulus. Multiphase heat transfer depends on the hydrodynamic behavior of the flow. There are very limited research results that can be found in the open literature for multiphase heat transfer in wellbore annuli. A mechanistic model of multiphase heat transfer is developed for different flow patterns of upward gas–liquid flow in vertical annuli. The required local flow parameters are predicted by use of the hydraulic model of steady-state multiphase flow in wellbore annuli recently developed by Yin et al. The modified heat-transfer model for single gas or liquid flow is verified by comparison with Manabe’s experimental results. For different flow patterns, it is compared with modified unified Zhang et al. model based on representative diameters.

## Introduction

As oil and gas development moves from land or shallow water to deep and ultradeep waters, multiphase flow occurs during production and transportation (Chen 2011). The flow normally occurs in horizontal, inclined, or vertical pipes and wells. Gas–liquid two-phase flow in an annulus can be found in a variety of practical situations. In high rate oil and gas production, it may be beneficial to flow fluids vertically through the annulus configuration between well tubing and casing. For surface facilities, some large, under-utilized flow lines can be converted to dual-service by putting a second pipe through the large line (i.e., flowing produced water in the inner line and gas in the annulus). During gas production, liquids may accumulate at the bottom of the gas wells during their later life. In order to remove or “unload” the undesirable liquids, a siphon tube is often installed inside the tubing string, which would form a gas–liquid two-phase flow in the annulus. Flow-assurance problems, such as hydrate blocking (Jamaluddin et al. 1991; Li et al. 2013; Wang et al. 2014, 2016) and wax deposition (Zhang et al. 2013; Bryan 2016; Theyab and Diaz 2016), are strongly associated with both the hydraulic and thermal behavior. For example, they are related to the fluid velocity, liquid fraction, slug characteristics, pressure gradient and convective-heat-transfer coefficients of different phase and flow patterns in multiphase flow. Therefore, multiphase hydrodynamics and heat transfer in an annulus need to be modeled properly to guide the design and operation of flow systems.

Compared to theoretical studies of multiphase hydrodynamics (Liu et al. 2007; Wang and Sun 2009), and multiphase heat transfer in pipe flow (Zheng et al. 2016; Gao et al. 2016; Karimi and Boostani 2016; Rushd and Sanders 2017), there are very limited research results in the open literature for multiphase heat transfer in wellbore annuli. Davis et al. (1979) obtained a model for calculating the local Nusselt numbers of stratified gas/liquid flow in turbulent liquid/turbulent gas conditions. The model was tested with heat-transfer experiments for air/water flow in a 63.5-mm inside diameter (ID) tube. Guo et al. (2017) established a mathematical model of heat transfer in a gas-drilling system, considering the flowing gas, formation fluid influx, Joule–Thomson cooling and entrained cuttings in the annular space. However, the multiphase flow effect on the heat transfer was not considered.

Shoham, et al. (1982) undertook experiments on heat-transfer for slug flow in a horizontal pipe. He found a substantial difference in heat-transfer coefficient existed between the top and bottom of the slug. Developing heat-transfer correlations of different flow patterns was the aim of most previous studies (Shah 1981). Twenty heat-transfer correlations were compared in Kim’s study (Kim et al. 1997). He collected the experimental data from the open literature and recommended the correlations for different flow patterns. However, the errors with the experimental results by Matzain (1999) were large. Later, a comprehensive mechanistic model about heat transfer in gas–liquid pipe flow was obtained (Manabe 2001). It was compared with the experimental data, and the performance was better. However, there were some inconsistencies in annular and slug flow. It needed to be modified.

**Fig. 1:**
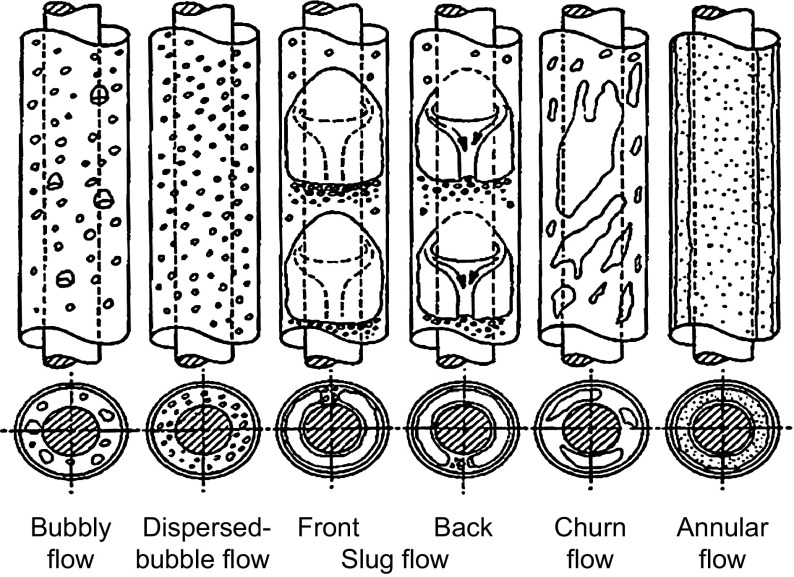
Flow patterns for upward vertical flow in an annulus (Caetano 1986)

Zhang et al. (2006) developed a unified model of multiphase heat transfer for different flow patterns of gas–liquid pipe flow at all inclinations – 90° to + 90° from the horizontal. The required local flow parameters were predicted by use of the unified hydrodynamic model for gas/liquid pipe flow developed by Zhang et al. (2003a, b). However, it is not fit for the gas–liquid flow in an annulus, because the flow patterns in annuli are different from pipe flow patterns, as seen in Fig. 1 (Caetano 1986). A new heat-transfer model for gas–liquid flow in vertical annuli needs to be established.

A hydraulic model was developed to predict flow patterns, liquid holdup and pressure gradients for steady-state gas–liquid flow in wellbore annuli (Yin et al. 2014). The major advantage of this model compared with previous mechanistic models is that it is developed based on the dynamics of slug flow, and the liquid-film zone is used as the control volume. The effects of the tubing liquid film, casing liquid film and the droplets in the gas core area on the mass and momentum transfers are considered. Multiphase heat transfer depends on the hydrodynamic behavior of the flow. The objective of this study is to develop a heat-transfer model for gas–liquid flow that is consistent with the hydrodynamic model in vertical wellbore annuli.

## Modeling

### Single-phase flow

**Fig. 2:**
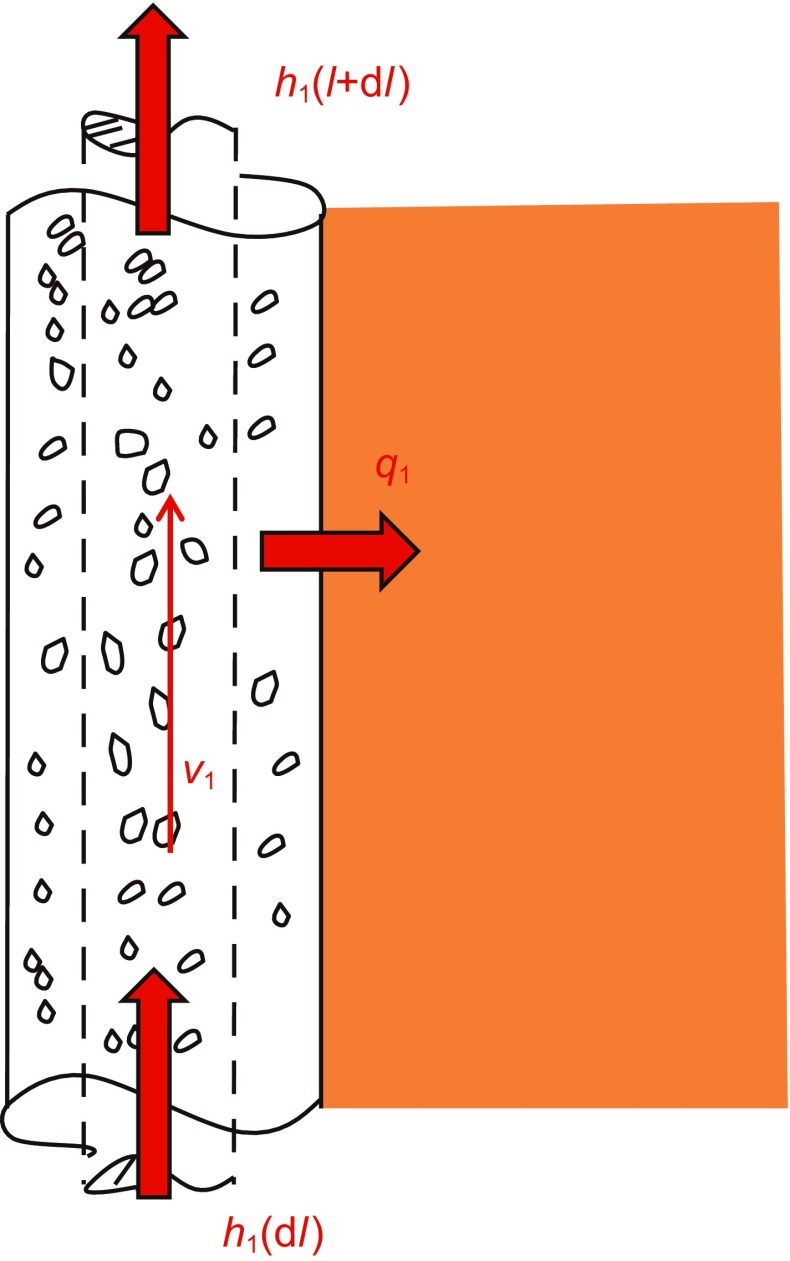
Heat transfer from the annulus to the formation

When fluids flow through an annulus, the surrounding temperature is cold, heat is lost from the fluids to the formation, resulting in a decline in temperature, as seen in Fig. 2. In the steady state, there is no heat loss from the annulus to the tube. For the fluids in the annulus, the conservation of mass is:

1 $$\frac{\text{d} }{{\text{d} l}}\left( {\rho_{\text{l}} v_{\text{l}} } \right) = 0$$

where *ρ*_l_ is the density of liquids, kg/cm^3^; *v*_l_ is the velocity of liquids in the annulus, m/s; d*l* is the length of the element.

The conservation of momentum is:

2 $$\frac{\text{d} }{{\text{d} l}}\left( {\rho_{\text{l}} v_{\text{l}}^{2} } \right) = - \frac{{{\text{d} p}}}{{\text{d} l}} - \rho_{\text{l}} g\sin \theta - \frac{\tau \pi d}{A}$$

where *p* is the unit pressure, MPa; *g* is the gravity acceleration, m/s^2^; *θ* is the inclination angle, °; *τ* is the friction; *d* is the annulus diameter, m; *A* is the cross area of the annulus, m^2^.

The energy equation is:

3 $$\frac{\text{d}}{{{\text{d}}l}}\left( {\rho_{\text{l}} v_{\text{l}} \left( {e + \frac{1}{2}v_{\text{l}}^{2} } \right)} \right) = - \frac{\text{d}}{{{\text{d}}l}}\left( {pv_{\text{l}} } \right) - \rho_{\text{l}} v_{\text{l}} g\sin \theta - \frac{{q_{1} }}{A}$$

where *e* is the internal energy of the unit, J/kg; *q*_1_ is the heat loss from the annulus to the formation, W.

Using the mass balance, we can reduce Eqs. (2) and (3) further:

4 $$\frac{{{\text{d}}p}}{{{\text{d}}l}} = - \rho_{\text{l}} v_{\text{l}} \frac{{{\text{d}}v_{\text{l}} }}{{{\text{d}}l}} - \rho_{\text{l}} g\sin \theta - \frac{\tau \pi d}{A}$$

5 $$\rho_{\text{l}} v_{\text{l}} \frac{\text{d}}{{{\text{d}}l}}\left( {e + \frac{p}{{\rho_{\text{l}} }}} \right) = - \rho_{\text{l}} v_{\text{l}} \frac{{{\text{d}}v_{\text{l}} }}{{{\text{d}}l}} - \rho_{\text{l}} v_{\text{l}} g\sin \theta - \frac{{q_{ 1} }}{A}$$

Or

6 $$\frac{{{\text{d}}h}}{{{\text{d}}l}} = - v_{\text{l}} \frac{{{\text{d}}v_{\text{l}} }}{{{\text{d}}l}} - g\sin \theta - \frac{{q_{1} }}{{A\rho_{\text{l}} v_{\text{l}} }} = - v_{\text{l}} \frac{{{\text{d}}v_{\text{l}} }}{{{\text{d}}l}} - g\sin \theta - \frac{{q_{1} }}{{w_{ 1} }}$$

where *h* is the enthalpy, J/kg; *w*_l_ is the mass flow rate of liquids in the annulus, kg/s.

Using the definition of the dimensionless temperature *T*_D_ proposed by Hasan and Kabir (1991), we may write an expression for heat transfer from the wellbore/formation interface to the formation as

7 $$q_{1} = \frac{{2\pi k_{\text{e}} \left( {T_{\text{wb}} - T_{\text{e}} } \right)}}{{T_{\text{D}} }}$$

where *T*_e_ is the formation temperature, °C; *T*_wb_ is the wellbore temperature, °C; *k*_e_ is the conductivity of formation, W/(m °C); the dimensionless temperature, defined by Hasan and Kabir (1991), *T*_D_, can be easily estimated from the following models.

$$T_{\text{D}} = \left\{ {\begin{array}{*{20}l} {1.1281\sqrt {t_{\text{D}} } \left( {1 - 0.3\sqrt {t_{\text{D}} } } \right),} \hfill &\quad {10^{ - 10} \le t_{\text{D}} \le 1.5} \hfill \\ {\left( {0.4063 + 0.5\ln t_{\text{D}} } \right)\left( {1 + \frac{0.6}{{t_{\text{D}} }}} \right),} \hfill &\quad {t_{\text{D}} > 1.5} \hfill \\ \end{array} } \right.$$

where *t*_D_ is the dimensionless flowing time,

$$t_{\text{D}} = \frac{t}{{r_{\text{w}}^{2} }}$$

where *t* is the flowing time, s; *r*_w_ is the wellbore radius, m.

The heat transfer from the annulus to the well wall, *q*_1_ may be expressed in terms of an overall heat coefficient (Hansan and Kabir 1994),

8 $$q_{1} = 2\pi r_{\text{co}} U_{\text{a}} \left( {T_{\text{a}} - T_{\text{wb}} } \right)$$

where *r*_co_ is the outside casing radius, m; *T*_a_ is the fluid temperature in the annulus, °C; *U*_a_ is the overall heat-transfer coefficient of the annulus, W/(m^2^ °C).

9 $$\frac{1}{{U_{\text{a}} }} = \frac{{r_{\text{co}} }}{{r_{\text{ci}} h_{\text{a}} }} + \frac{{r_{\text{co}} \ln \left( {{{r_{\text{co}} } \mathord{\left/ {\vphantom {{r_{\text{co}} } {r_{\text{ci}} }}} \right. \kern-0pt} {r_{\text{ci}} }}} \right)}}{{k_{\text{cas}} }} + \frac{{r_{\text{co}} \ln \left( {{{r_{\text{w}} } \mathord{\left/ {\vphantom {{r_{\text{w}} } {r_{\text{co}} }}} \right. \kern-0pt} {r_{\text{co}} }}} \right)}}{{k_{\text{cem}} }}$$

where *r*_ci_ is the inside casing radius, m; *h*_a_ is the convective-heat-transfer coefficient of fluids in the annulus, W/(m^2^ °C); *k*_cas_ is the conductivity of the casing wall, W/(m °C); *k*_cem_ is the conductivity of cement, W/(m °C).

Combining Eqs. (7) and (8) gives,

$$q_{1} = \frac{{2\pi k_{\text{e}} \left( {T_{\text{wb}} - T_{\text{e}} } \right)}}{{T_{\text{D}} }} = 2\pi r_{\text{co}} U_{\text{a}} \left( {T_{\text{a}} - T_{\text{wb}} } \right)$$

10 $$q_{1} = \frac{{w_{\text{l}} c_{\text{p}} }}{{A^{\prime}}}\left( {T_{\text{a}} - T_{\text{e}} } \right)$$

where *c*_p_ is the fluid heat capacity at the constant pressure in the annulus, J/(kg °C).

$$A^{\prime} = \frac{{w_{ 1} c_{\text{p}} }}{2\pi }\left[ {\frac{{k_{\text{e}} + r_{\text{co}} U_{\text{a}} T_{\text{D}} }}{{r_{\text{co}} U_{\text{a}} k_{\text{e}} }}} \right]$$

where $A^{\prime}$ is the local parameter defined in Eq. (10).

Combining Eqs. (6), (8) and (10) yields,

11 $$\frac{{{\text{d}}h}}{{{\text{d}}l}} = - v_{\text{l}} \frac{{{\text{d}}v_{\text{l}} }}{{{\text{d}}l}} - g\sin \theta - \frac{{c_{\text{p}} }}{{A^{\prime}}}\left( {T_{\text{a}} - T_{\text{e}} } \right)$$

The enthalpy gradient can be written in terms of the temperature and pressure gradients:

12 $$\frac{{{\text{d}}h}}{{{\text{d}}l}} = c_{\text{p}} \frac{{{\text{d}}T}}{{{\text{d}}l}} - \eta_{\text{l}} c_{\text{p}} \frac{{{\text{d}}p}}{{{\text{d}}l}}$$

where *η*_l_ is the fluid Joule–Thomson coefficient in the annulus, °C/MPa.

Combining Eqs. (11) and (12) gives

13 $$\frac{{{\text{d}}T_{\text{a}} }}{{{\text{d}}l}} + \frac{1}{{A^{\prime}}}\left( {T_{\text{a}} - T_{\text{e}} } \right) + \frac{1}{{c_{\text{p}} }}\left( {v_{\text{l}} \frac{{{\text{d}}v_{\text{l}} }}{{{\text{d}}l}} + g\sin \theta - \eta_{\text{l}} c_{\text{p}} \frac{{{\text{d}}p}}{{{\text{d}}l}}} \right) = 0$$

Defining a dimensionless parameter *Φ*_a_, as

$$\varPhi_{\text{a}} = {{\left( {\rho_{\text{l}} v_{\text{l}} \frac{{{\text{d}}v_{\text{l}} }}{{{\text{d}}l}} + \rho_{\text{l}} g\sin \theta - \rho_{\text{l}} \eta_{\text{l}} c_{\text{p}} \frac{{{\text{d}}p}}{{{\text{d}}l}}} \right)} \mathord{\left/ {\vphantom {{\left( {\rho_{\text{l}} v_{\text{l}} \frac{{{\text{d}}v_{\text{l}} }}{{{\text{d}}l}} + \rho_{\text{l}} g\sin \theta - \rho_{\text{l}} \eta_{\text{l}} c_{\text{p}} \frac{{{\text{d}}p}}{{{\text{d}}l}}} \right)} {\frac{{{\text{d}}p}}{{{\text{d}}l}}}}} \right. \kern-0pt} {\frac{{{\text{d}}p}}{{{\text{d}}l}}}}$$

We can write Eq. (13) as

14 $$\frac{{{\text{d}}T_{\text{a}} }}{{{\text{d}}l}} + \frac{1}{{A^{\prime}}}\left( {T_{\text{a}} - T_{\text{e}} } \right) + \frac{1}{{\rho_{\text{l}} c_{\text{p}} }}\frac{{{\text{d}}p}}{{{\text{d}}l}}\varPhi_{\text{a}} = 0$$

Equation (14) is the energy conservation equation of fluids in the annulus.

We can write Eq. (14) as

15 $$\frac{{{\text{d}}T_{\text{a}} }}{{{\text{d}}l}} + \frac{1}{{A^{\prime}}}T_{\text{a}} = \frac{1}{{A^{\prime}}}T_{\text{e}} - \frac{1}{{\rho_{\text{l}} c_{\text{p}} }}\frac{{{\text{d}}p}}{{{\text{d}}l}}\varPhi_{\text{a}}$$

Up to this point, only mathematical manipulations have been done to the enthalpy equation, and the analysis has been carried out rigorously without simplification. Now if Eq. (15) is used for an onshore production well, then assuming the surrounding formation temperature is a linear function of depth, it can be expressed as

16 $$T_{\text{e}} = T_{\text{ei}} - g_{\text{e}} L\sin \theta$$

where *T*_ei_ is the temperature of formation at wellbore intake, °C; *g*_e_ is the formation thermal gradient, °C/100 m; *L* is the depth, m.

If Eq. (15) is used for the riser of an offshore production well, then the surrounding sea temperature is not a linear function of depth. It will be calculated according to the actual environment (Wang and Sun 2009).

If, for a certain segment of the wellbore, *U*_a_, *c*_p_, *η*_l_, *g*_e_, *θ*, *v*_l_d*v*_l_/d*l* and d*p*/d*l* can be approximately constants, combining Eqs. (15) and (16) and integrating, yields an explicit equation for the temperature:

17 $$\begin{aligned} T_{\text{a}} & = \left( {T_{\text{ei}} - g_{\text{e}} L\sin \theta } \right) + \left( {T_{\text{i}} - T_{\text{ei}} } \right)\exp \left( { - {L \mathord{\left/ {\vphantom {L {A^{\prime } }}} \right. \kern-0pt} {A^{\prime } }}} \right) + g_{\text{e}} A^{\prime } \sin \theta \\ & \quad \times \left[ {1 - \exp \left( { - {L \mathord{\left/ {\vphantom {L {A^{\prime } }}} \right. \kern-0pt} {A^{\prime } }}} \right)} \right] + \frac{1}{{\rho c_{\text{p}} }}\frac{{{\text{d}}p}}{{{\text{d}}L}}\frac{\varPhi }{{A^{\prime } }}\left[ {1 - \exp \left( { - {L \mathord{\left/ {\vphantom {L {A^{\prime } }}} \right. \kern-0pt} {A^{\prime } }}} \right)} \right] \\ \end{aligned}$$

### Bubbly flow and dispersed-bubble flow

In bubbly flow and dispersed-bubble flow, the gas holdup is small and the gas superficial velocity is low, the gas phase is distributed as small discrete bubbles in a continuous liquid phase. So bubbly flow and dispersed-bubble flow can be treated as pseudo-single-phase flow. The fluid physical properties are adjusted based on the liquid holdup. Zhang et al. correction (2006) for bubbly flow will be modified based on “hydraulic diameter”.

17 $$d_{\text{R}} = d_{\text{ci}} - d_{\text{tuo}}$$

where *d*_R_ is the hydraulic diameter, m; *d*_ci_ is the inside diameter of casing, m; *d*_tuo_ is the outside diameter of tubing, m.

Then the convective-heat-transfer coefficient of Eq. (9) for bubbly or dispersed-bubbly flow is obtained from

18 $$h_{\text{ab}} = \frac{{N_{\text{Nu}}^{\text{m}} k_{\text{m}} }}{{d_{\text{R}} }}$$

where *k*_m_ is the conductivity of the mixture, W/(m °C),

$$k_{\text{m}} = \left( {1 - H_{\text{l}} } \right)k_{\text{g}} + H_{\text{l}} k_{\text{l}}$$

where *H*_l_ is the liquid hold up; *k*_l_ is the conductivity of liquid, W/(m °C); *k*_g_ is the conductivity of gas, W/(m °C).

$N_{\text{Nu}}^{\text{m}}$ is the mixture Nusselt number (Zhang et al. 2006).

19 $$N_{\text{Nu}}^{\text{m}} = \frac{{\left( {\frac{{f_{\text{m}} }}{2}} \right)N_{\text{Re}}^{\text{m}} N_{\Pr }^{\text{m}} }}{{1.07 + 12.7\sqrt {\frac{{f_{\text{m}} }}{2}} \left( {N_{\Pr }^{{{\text{m}}^{{{2 \mathord{\left/ {\vphantom {2 3}} \right. \kern-0pt} 3}}} }} - 1} \right)}}\left( {\frac{{\mu_{\text{l}} }}{{\mu_{\text{m}} }}} \right)$$

where *f*_m_ is the friction factor of the mixture; *μ*_l_ is the viscosity of liquid, mPa s; *μ*_m_ is the viscosity of the mixture, mPa s; *μ*_g_ is the viscosity of gas, mPa s,

$$\mu_{\text{m}} = \left( {1 - H_{\text{l}} } \right)\mu_{\text{g}} + H_{\text{l}} \mu_{\text{l}}$$

$N_{\text{Re}}^{\text{m}}$ and $N_{ \Pr }^{\text{m}}$ is the Reynolds number and Prandtl number of the mixture.

$$N_{\text{Re}}^{\text{m}} = \frac{{\rho_{\text{m}} v_{\text{m}} d_{\text{R}} }}{{\mu_{\text{m}} }}$$

$$N_{\Pr }^{\text{m}} = \frac{{c_{\text{pm}} \mu_{\text{m}} }}{{k_{\text{m}} }}$$

where *ρ*_m_ is the density of the mixture, kg/m^3^; *v*_m_ is the velocity of the mixture, m/s; *c*_pm_ is the specific heat of the mixture, J/(kg °C),

$$c_{\text{pm}} = \left( {1 - H_{\text{l}} } \right)c_{\text{pg}} + H_{\text{l}} c_{\text{pl}}$$

where *c*_pl_ is the specific heat of liquid, J/(kg °C); *c*_pg_ is the specific heat of gas, J/(kg °C).

So the associated parameters will be modified,

$$\frac{1}{{U_{\text{ab}} }} = \frac{{r_{\text{co}} }}{{r_{\text{ci}} h_{\text{ab}} }} + \frac{{r_{\text{co}} \ln \left( {{{r_{\text{co}} } \mathord{\left/ {\vphantom {{r_{\text{co}} } {r_{\text{ci}} }}} \right. \kern-0pt} {r_{\text{ci}} }}} \right)}}{{k_{\text{cas}} }} + \frac{{r_{\text{co}} \ln \left( {{{r_{\text{w}} } \mathord{\left/ {\vphantom {{r_{\text{w}} } {r_{\text{co}} }}} \right. \kern-0pt} {r_{\text{co}} }}} \right)}}{{k_{\text{cem}} }}$$

$$A^{\prime}_{\text{b}} = \frac{{w_{\text{m}} c_{\text{pm}} }}{2\pi }\left[ {\frac{{k_{\text{e}} + r_{\text{co}} U_{\text{ab}} T_{\text{D}} }}{{r_{\text{co}} U_{\text{ab}} k_{\text{e}} }}} \right]$$

$$\varPhi_{\text{ab}} = {{\left( {\rho_{\text{m}} v_{\text{m}} \frac{{{\text{d}}v_{\text{m}} }}{{{\text{d}}l}} + \rho_{\text{m}} g\sin \theta - \rho_{\text{m}} \eta_{\text{m}} c_{\text{pm}} \frac{{{\text{d}}p}}{{{\text{d}}l}}} \right)} \mathord{\left/ {\vphantom {{\left( {\rho_{\text{m}} v_{\text{m}} \frac{{{\text{d}}v_{\text{m}} }}{{{\text{d}}l}} + \rho_{\text{m}} g\sin \theta - \rho_{\text{m}} \eta_{\text{m}} c_{\text{pm}} \frac{{{\text{d}}p}}{{{\text{d}}l}}} \right)} {\frac{{{\text{d}}p}}{{{\text{d}}l}}}}} \right. \kern-0pt} {\frac{{{\text{d}}p}}{{{\text{d}}l}}}}$$

where the subscript b represents in bubble flow or dispersed-bubble flow; the subscript m represents the mixture properties of gas and liquid, $A^{\prime}_{\text{b}}$ is the local parameter defined in Eq. (20).

Equation (15) can be written as

20 $$\frac{{{\text{d}}T_{\text{a}} }}{{{\text{d}}l}} + \frac{1}{{A^{\prime}_{\text{b}} }}T_{\text{a}} = \frac{1}{{A^{\prime}_{\text{b}} }}T_{\text{e}} - \frac{1}{{\rho_{\text{m}} c_{\text{pm}} }}\frac{{{\text{d}}p}}{{{\text{d}}l}}\varPhi_{\text{ab}}$$

Then Eq. (20) can be used in the heat transfer in bubble flow and dispersed-bubble flow based on the modified convective-heat-transfer coefficient *h*_ab_.

### Annular flow

**Fig. 3:**
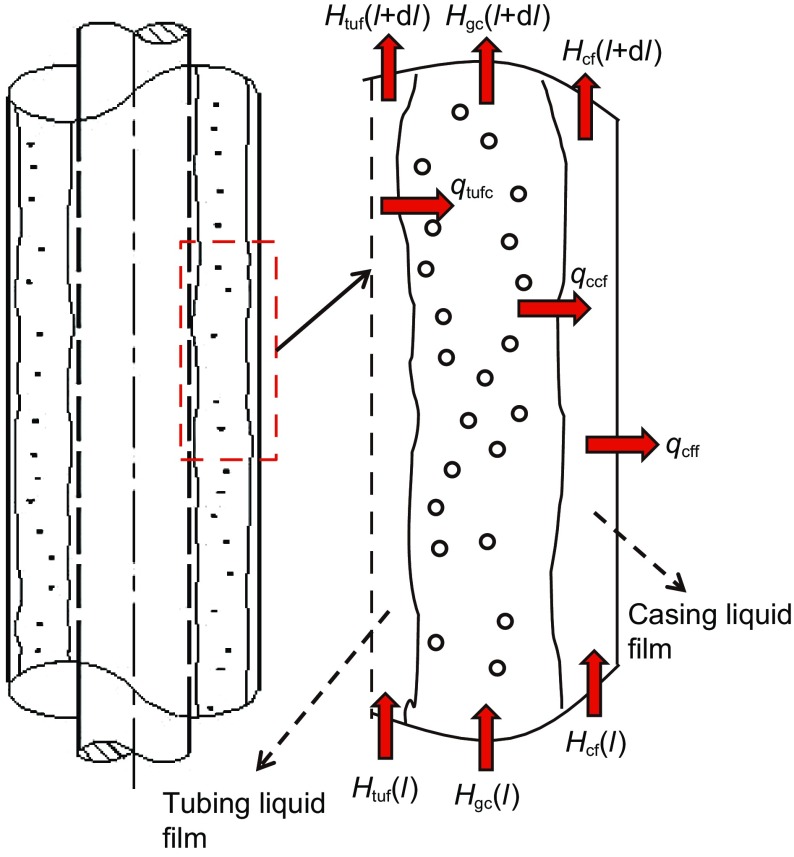
Control volume and temperatures in annular flow (*q*_tufc_ is the heat transfer from the tubing film to the gas core, W; *q*_ccf_ is the heat transfer from the gas core to the casing film, W; *q*_cff_ is the heat transfer from the casing film to the formation, W)

As shown in Fig. 3, there are tubing and casing films in annular flow in annuli, which is different from the annular flow in pipes. Assume there are no temperature and heat-transfer exchanges in the vertical direction. The temperature and heat changes are only caused by the heat transfer in the radial direction. Heat distribution in annuli includes three parts: in casing film, tubing film and gas core. The heat-transfer models in annuli are obtained by a method similar to that above.

For the gas core, the energy conservation equation is as follows,

21 $$\frac{{{\text{d}}T_{\text{gc}} }}{{{\text{d}}l}} + \frac{1}{{A^{\prime}_{\text{gc}} }}\left( {T_{\text{gc}} - T_{\text{cf}} } \right) - \frac{1}{{B^{\prime}_{\text{gc}} }}\left( {T_{\text{tuf}} - T_{\text{gc}} } \right) + \frac{1}{{\rho_{\text{gc}} c_{\text{pgc}} }}\frac{{{\text{d}}p}}{{{\text{d}}l}}\varPhi_{\text{gc}} = 0$$

where *T*_gc_ is the temperature of the gas core, °C; *T*_cf_ is the temperature of the casing film, °C; *T*_tuf_ is the temperature of the tubing film, °C; *c*_pgc_ is the specific heat of the gas core, J/(kg °C); $A^{\prime}_{\text{gc}}$, $B^{\prime}_{\text{gc}}$, *Φ*_gc_ are the local parameters defined as follows:

$$A^{\prime}_{\text{gc}} = \frac{{w_{\text{gc}} c_{\text{pgc}} }}{{2\pi \left( {r_{\text{ci}} - \delta_{\text{c}} } \right)U_{\text{gc}} }}$$

$$B^{\prime}_{\text{gc}} = \frac{{w_{\text{gc}} c_{\text{pgc}} }}{{2\pi \left( {r_{\text{tuo}} + \delta_{\text{tu}} } \right)U_{\text{tuf}} }}$$

$$\varPhi_{\text{gc}} = {{\left( {\rho_{\text{gc}} v_{\text{gc}} \frac{{{\text{d}}v_{\text{gc}} }}{{{\text{d}}l}} + \rho_{\text{gc}} g\sin \theta - \rho_{\text{gc}} \eta_{\text{gc}} c_{\text{pgc}} \frac{{{\text{d}}p}}{{{\text{d}}l}}} \right)} \mathord{\left/ {\vphantom {{\left( {\rho_{\text{gc}} v_{\text{gc}} \frac{{{\text{d}}v_{\text{gc}} }}{{{\text{d}}l}} + \rho_{\text{gc}} g\sin \theta - \rho_{\text{gc}} \eta_{\text{gc}} c_{\text{pgc}} \frac{{{\text{d}}p}}{{{\text{d}}l}}} \right)} {\frac{{{\text{d}}p}}{{{\text{d}}l}}}}} \right. \kern-0pt} {\frac{{{\text{d}}p}}{{{\text{d}}l}}}}$$

where *w*_gc_ is the mass flow rate of the gas core, kg/s; *c*_pgc_ is the specific heat of the gas core, J/(kg °C); *v*_gc_ is the velocity of the gas core, m/s; *ρ*_gc_ is the density of the gas core, kg/m^3^; *η*_gc_ is the Joule–Thomson coefficient of the gas core, °C/MPa; *U*_gc_ is the overall heat-transfer coefficient of the gas core, W/(m^2^ °C), and defined as

$$\frac{1}{{U_{\text{gc}} }} = \frac{{r_{\text{ci}} - \delta_{\text{c}} }}{{\left( {r_{\text{tuo}} + \delta_{\text{tu}} } \right)h_{\text{gc}} }}$$

where *r*_tuo_ is the outside radius of the tubing, m; *δ*_tu_ is the thickness of the tubing film, m; *δ*_c_ is the thickness of the casing film, m; *h*_gc_ is the convective-heat-transfer coefficient of the gas core, W/(m^2^ °C).

*U*_tuf_ is the overall heat-transfer coefficient of the tubing film, W/(m^2^ °C), and defined as

$$\frac{1}{{U_{\text{tuf}} }} = \frac{{r_{\text{tuo}} + \delta_{\text{tu}} }}{{r_{\text{tuo}} h_{\text{tuf}} }} + \frac{{\left( {r_{\text{tuo}} + \delta_{\text{tu}} } \right)\ln \left( {{{\left( {r_{\text{tuo}} + \delta_{\text{tu}} } \right)} \mathord{\left/ {\vphantom {{\left( {r_{\text{tuo}} + \delta_{\text{tu}} } \right)} {r_{\text{tuo}} }}} \right. \kern-0pt} {r_{\text{tuo}} }}} \right)}}{{k_{\text{tu}} }}$$

where *h*_tuf_ is the convective-heat-transfer coefficient of the tubing film, W/(m^2^ °C); *k*_tu_ is the conductivity of the tubing wall, W/(m °C).

For the tubing film, the energy conservation equation is as follows,

22 $$\frac{{{\text{d}}T_{\text{tuf}} }}{{{\text{d}}l}} + \frac{{w_{\text{gc}} c_{\text{pgc}} }}{{w_{\text{tuf}} c_{\text{ptuf}} B^{\prime}_{\text{gc}} }}\left( {T_{\text{tuf}} - T_{\text{gc}} } \right) + \frac{1}{{\rho_{\text{tuf}} c_{\text{ptuf}} }}\frac{{{\text{d}}p}}{{{\text{d}}l}}\varPhi_{\text{tuf}} = 0$$

where *w*_tuf_ is the mass flow rate of the liquid tubing film, kg/s; *ρ*_tuf_ is the density of the liquid tubing film, kg/m^3^; *c*_ptuf_ is the specific heat of the liquid tubing film, J/(kg °C); *Φ*_tuf_ is the local constant and defined as follows:

$$\varPhi_{\text{tuf}} = {{\left( {\rho_{\text{tuf}} v_{\text{tuf}} \frac{{{\text{d}}v_{\text{tuf}} }}{{{\text{d}}l}} + \rho_{\text{tuf}} g\sin \theta - \rho_{\text{tuf}} \eta_{\text{tuf}} c_{\text{ptuf}} \frac{{{\text{d}}p}}{{{\text{d}}l}}} \right)} \mathord{\left/ {\vphantom {{\left( {\rho_{\text{tuf}} v_{\text{tuf}} \frac{{{\text{d}}v_{\text{tuf}} }}{{{\text{d}}l}} + \rho_{\text{tuf}} g\sin \theta - \rho_{\text{tuf}} \eta_{\text{tuf}} c_{\text{ptuf}} \frac{{{\text{d}}p}}{{{\text{d}}l}}} \right)} {\frac{{{\text{d}}p}}{{{\text{d}}l}}}}} \right. \kern-0pt} {\frac{{{\text{d}}p}}{{{\text{d}}l}}}}$$

where *v*_tuf_ is the velocity of the liquid tubing film, m/s; *η*_tuf_ is the Joule–Thomson coefficient of the liquid tubing film, °C/MPa.

For the casing film, the energy conservation equation is as follows,

23 $$\frac{{{\text{d}}T_{\text{cf}} }}{{{\text{d}}l}} + \frac{1}{{C^{\prime}_{\text{cf}} }}\left( {T_{\text{cf}} - T_{\text{e}} } \right) - \frac{{w_{\text{gc}} c_{\text{pgc}} }}{{w_{\text{cf}} c_{\text{pcf}} A^{\prime}_{\text{gc}} }}\left( {T_{\text{gc}} - T_{\text{cf}} } \right) + \frac{1}{{\rho_{\text{cf}} c_{\text{pcf}} }}\frac{{{\text{d}}p}}{{{\text{d}}l}}\varPhi_{\text{cf}} = 0$$

where *w*_cf_ is the mass flow rate of liquid casing film, kg/s; *ρ*_cf_ is the density of liquid casing film, kg/m^3^; *c*_pcf_ is the specific heat of liquid casing film, J/(kg °C); $C^{\prime}_{\text{cf}}$ is the local parameter defined in Eq. (23),

$$C^{\prime}_{\text{cf}} = \frac{{w_{\text{cf}} c_{\text{pcf}} }}{2\pi }\left[ {\frac{{k_{\text{e}} + r_{\text{co}} U_{\text{cf}} T_{\text{D}} }}{{r_{\text{co}} U_{\text{cf}} k_{\text{e}} }}} \right]$$

*U*_cf_ is the overall heat-transfer coefficient of casing film, W/(m^2^ °C), and defined as

$$\frac{1}{{U_{\text{cf}} }} = \frac{{r_{\text{ci}} }}{{\left( {r_{\text{ci}} - \delta_{\text{c}} } \right)h_{\text{cf}} }} + \frac{{r_{\text{co}} \ln \left( {{{r_{\text{co}} } \mathord{\left/ {\vphantom {{r_{\text{co}} } {r_{\text{ci}} }}} \right. \kern-0pt} {r_{\text{ci}} }}} \right)}}{{k_{\text{cas}} }} + \frac{{r_{\text{co}} \ln \left( {{{r_{\text{w}} } \mathord{\left/ {\vphantom {{r_{\text{w}} } {r_{\text{co}} }}} \right. \kern-0pt} {r_{\text{co}} }}} \right)}}{{k_{\text{cem}} }}$$

where *h*_cf_ is the convective-heat-transfer coefficient of casing film, W/(m^2^ °C).

*Φ*_cf_ is the local parameter defined in Eq. (23),

$$\varPhi_{\text{cf}} = {{\left( {\rho_{\text{cf}} v_{\text{cf}} \frac{{{\text{d}}v_{\text{cf}} }}{{{\text{d}}l}} + \rho_{\text{cf}} g\sin \theta - \rho_{\text{cf}} \eta_{\text{cf}} c_{\text{pcf}} \frac{{{\text{d}}p}}{{{\text{d}}l}}} \right)} \mathord{\left/ {\vphantom {{\left( {\rho_{\text{cf}} v_{\text{cf}} \frac{{{\text{d}}v_{\text{cf}} }}{{{\text{d}}l}} + \rho_{\text{cf}} g\sin \theta - \rho_{\text{cf}} \eta_{\text{cf}} c_{\text{pcf}} \frac{{{\text{d}}p}}{{{\text{d}}l}}} \right)} {\frac{{{\text{d}}p}}{{{\text{d}}l}}}}} \right. \kern-0pt} {\frac{{{\text{d}}p}}{{{\text{d}}l}}}}$$

where *v*_cf_ is the velocity of the liquid casing film, m/s; *η*_cf_ is the Joule–Thomson coefficient of the liquid casing film, °C/MPa.

Equations (21)–(23) are the heat-transfer models for gas–liquid annular flow in annuli. Then the fluid temperature in the wellbore is calculated by a weighted average method based on holdup.

24 $$T_{\text{ta}} = H_{\text{tuf}} T_{\text{tuf}} + \left( {1 - H_{\text{tuf}} - H_{\text{cf}} } \right)T_{\text{gc}} + H_{\text{cf}} T_{\text{cf}}$$

where *H*_tuf_ is the holdup of the liquid tubing film; *H*_cf_ is the holdup of the liquid casing film.

The convective-heat-transfer coefficients for the casing film, the tubing film and gas core are obtained by the following equations

$$h_{\text{tuf}} = \frac{{N_{\text{Nu}}^{\text{tuf}} k_{\text{lf}} }}{{d_{\text{tufR}} }},\quad h_{\text{gc}} = \frac{{N_{\text{Nu}}^{\text{gc}} k_{\text{gc}} }}{{d_{\text{gcR}} }},\quad h_{\text{cf}} = \frac{{N_{\text{Nu}}^{\text{cf}} k_{\text{lf}} }}{{d_{\text{cfR}} }}$$

where *k*_lf_, *k*_gc_ are the thermal conductivities of the liquid film and gas core, W/(m °C); *d*_tufR_, *d*_cfR_, *d*_gcR_ are the “hydraulic diameter” of the tubing film, the casing film and gas core, m.

$$d_{\text{tufR}} = \left( {r_{\text{tuo}} + \delta_{\text{tu}} } \right) - r_{\text{tuo}} = \delta_{\text{tu}} 
\quad d_{\text{cfR}} = r_{\text{ci}} - \left( {r_{\text{ci}} - \delta_{\text{c}} } \right) = \delta_{\text{c}}
\quad d_{\text{gcR}} = \left( {r_{\text{ci}} - \delta_{\text{c}} } \right) - \left( {r_{\text{tuo}} + \delta_{\text{tu}} } \right)$$

The Nusselt numbers for the liquid film and gas core are calculated by using the correlations for single-phase convective heat transfer. The Petukhov correlation (1970) is used for turbulent liquid-film flow:

25 $$N_{\text{Nu}}^{\text{if}} = \frac{{\left( {\frac{{f_{\text{if}} }}{2}} \right)N_{\text{Re}}^{\text{if}} N_{\Pr }^{\text{if}} }}{{1.07 + 12.7\sqrt {\frac{{f_{\text{if}} }}{2}} \left( {\left( {N_{\Pr }^{\text{if}} } \right)^{{{2 \mathord{\left/ {\vphantom {2 3}} \right. \kern-0pt} 3}}} - 1} \right)}}\left( {\frac{{\mu_{\text{l}} }}{{\mu_{\text{m}} }}} \right)^{0.25}$$

where *i* is tubing film or casing film, *i* = tu or c. *f*_if_ is the friction factor at the wall in contact with the liquid film.

$$N_{\text{Re}}^{\text{if}} = \frac{{\rho_{\text{l}} v_{\text{if}} d_{\text{ifR}} }}{{\mu_{\text{l}} }}\quad N_{\Pr }^{\text{if}} = \frac{{c_{\text{pl}} \mu_{\text{ifR}} }}{{k_{\text{l}} }}$$

The Dittus and Boelter correlation (1985) is used for turbulent gas core flow,

26 $$N_{\text{Nu}}^{\text{gc}} = 0.023\left( {N_{\text{Re}}^{\text{gc}} } \right)^{0.8} \left( {N_{\Pr }^{\text{gc}} } \right)^{0.33}$$

For fully developed laminar flows of the liquid film and gas core, the Nusselt number approaches a constant value. According to Zhang et al. (2006), the Nusselt numbers for fully developed laminar flows are calculated by,

27 $$N_{\text{Nu}}^{\text{if}} = 3.657 + \frac{7.541 - 3.657}{0.5}\left( {0.5 - \frac{{\delta_{\text{i}} }}{{2\sqrt {r_{\text{ci}}^{2} - r_{\text{tuo}}^{2} } }}} \right)$$

28 $$N_{\text{Nu}}^{\text{gc}} = 3.657$$

The associated hydraulic parameters are calculated by the Yin et al. model (Yin et al. 2014), such as the thickness of the liquid film, *δ*_c_ and *δ*_tu_, the hold up, liquid and gas physical properties .

### Slug flow

#### Film region

The flow characters of the film region are similar to the annular flow. The difference is that the gas core with liquid droplets changes into the Taylor bubble. So, the overall heat-transfer coefficients of Eqs. (21) and (23) should change from *U*_gc_ to *U*_T_. Then the heat transfer of the film region can be calculated.

29 $$\frac{1}{{U_{\text{T}} }} = \frac{1}{{h_{\text{T}} }}$$

where *U*_T_ is the overall heat-transfer coefficient of the Taylor bubble, W/(m^2^ °C); *h*_T_ is the convective-heat-transfer coefficient of the Taylor bubble, W/(m^2^ °C).

30 $$h_{\text{T}} = \frac{{N_{\text{Nu}}^{\text{T}} k_{\text{T}} }}{{d_{\text{TR}} }}$$

where *k*_T_ is the thermal conductivities of the Taylor bubble, W/(m °C); *d*_TR_ is the “representative diameter” of the Taylor bubble.

$$d_{\text{TR}} = 2\sqrt {\left( {r_{\text{ci}} - \delta_{\text{c}} } \right)^{2} - \left( {r_{\text{tuo}} + \delta_{\text{tu}} } \right)^{2} }$$

#### Slug region

There are small discrete bubbles in a continuous liquid phase. The flow characters are similar to bubbly flow. So, the heat transfer can be calculated by the bubbly flow model.

#### Slug unit

The fluid temperature in the wellbore is calculated by the weighted average method based on holdup.

31 $$T_{\text{s}} = \frac{{\left[ {H_{\text{tuf}} T_{\text{tuf}} + \left( {1 - H_{\text{tuf}} - H_{\text{cf}} } \right)T_{\text{T}} + H_{\text{cf}} T_{\text{cf}} } \right]l_{\text{F}} + T_{\text{ls}} l_{\text{s}} }}{{l_{\text{U}} }}$$

where *T*_s_ is the temperature of the fluid in slug flow, °C; *T*_ls_ is the temperature of the fluid in the slug unit, °C; *T*_T_ is the temperature of the Taylor bubble, °C; *l*_F_ is the length of the liquid film, m; *l*_s_ is the length of the slug unit, m; *l*_U_ is the length of the whole slug, m.

## Solution procedure

**Fig. 4:**
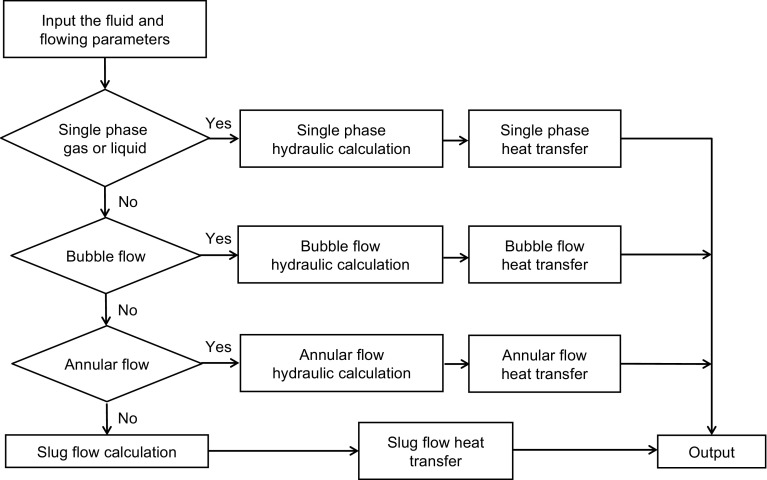
Overall flowchart for present model

Figure 4 shows the overall solution flowchart for the present model. First, the parameters of fluid properties are provided. Then the flow pattern is determined based on the input variables and then all the flow conditions, such as flow pattern, liquid holdups, local fluid velocities of the liquid film and gas core, and slug characteristics, are predicted by use of the hydraulic model of steady-state multiphase flow in wellbore annuli recently developed by Yin et al. (2014).

**Fig. 5:**
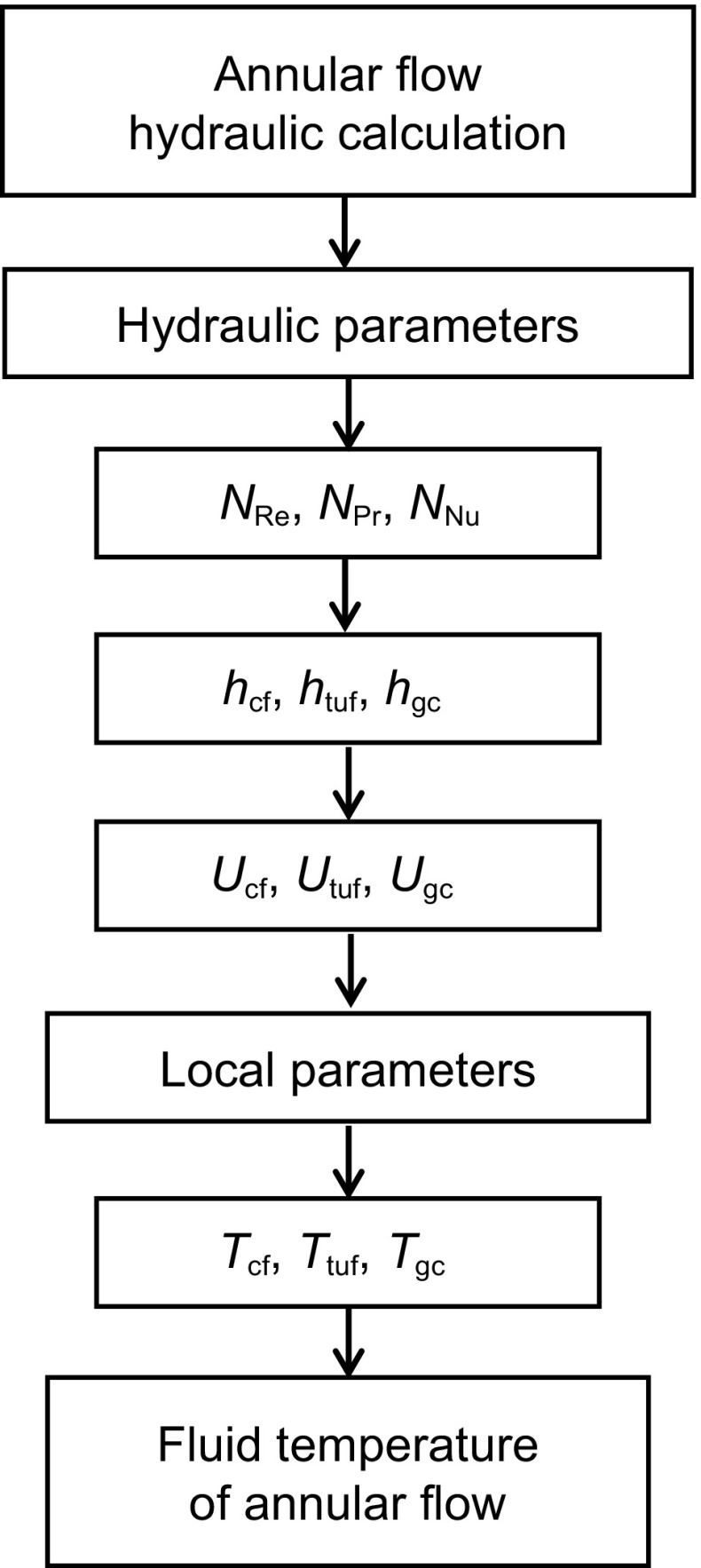
Flowchart for present annular flow heat-transfer model

Based on flow patterns, further calculations are performed in different subroutines. If the flow pattern is single-phase flow, single-phase heat-transfer calculation will be performed; if the flow pattern is bubble or dispersed-bubble flow, the corresponding hydraulic model and heat-transfer model will be called for calculation. For annular flow, corresponding hydraulic and heat-transfer calculations will be made. For slug flow, corresponding hydraulic and heat-transfer calculations will be made, as seen in Fig. 4. Figure 5 is the flowchart for the present annular flow heat-transfer model.

## Comparisons with the unified Zhang model

The heat transfer of single-phase gas or liquid flow can be calculated by the present model if the thermal conductivity of the tubing wall is infinite. The composition of simulated fluids is shown in Tables 1 and 2. The simulated annulus outer diameter is 76.2 mm, and the inner diameter is 42.2 mm. The basic parameters are shown in Table 3.

**Table 1:** Composition of natural gas

Components	Molar fraction, %
N_2_	1.82
CO_2_	0.65
C_1_	93.83
C_2_	2.98
C_3_	0.59
*i*-C_4_	0.08
*n*-C_4_	0.05

**Table 2:**
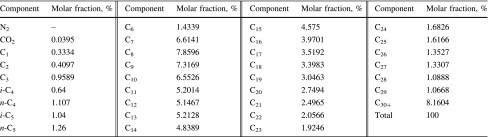
Composition of oil

**Table 3:**
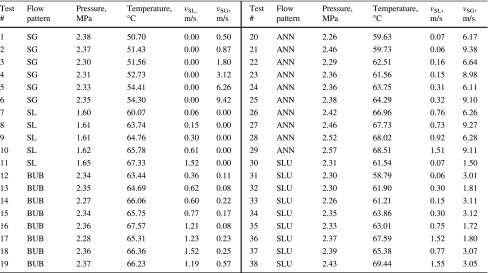
Basic parameters for the simulation

*SG* single-gas flow, *SL* single-liquid flow, *BUB* bubble flow, *ANN* annular flow, *SLU* slug flow

**Fig. 6:**
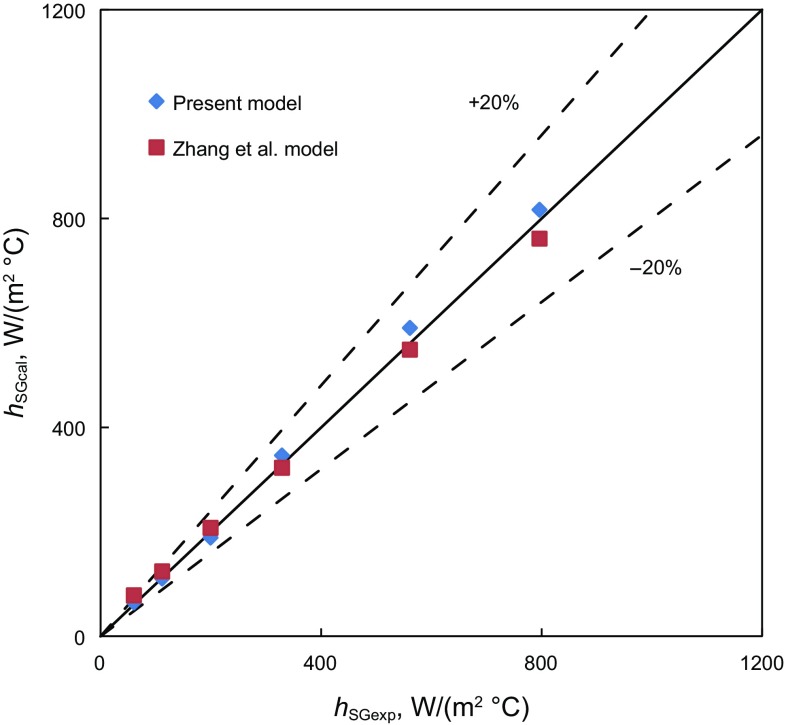
Comparison of single-phase gas flow model simulations and measured convective-heat-transfer coefficient

**Fig. 7:**
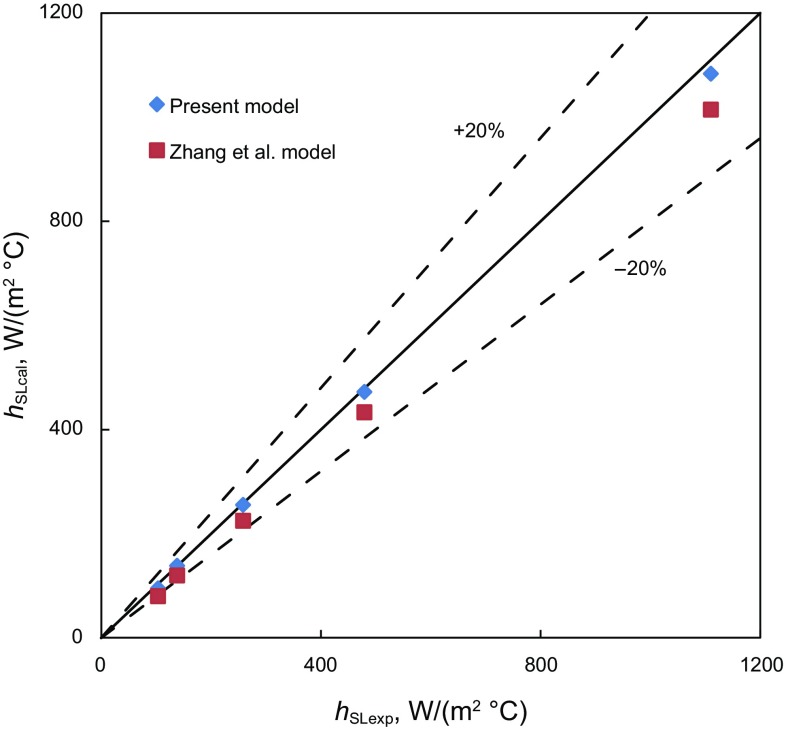
Comparison of single-phase liquid flow model simulations and measured convective-heat-transfer coefficients

Figures 6 and 7 show comparisons between the simulations of new models and experimental measurements (Manabe 2001) of the convective-heat-transfer coefficients for single-phase gas and liquid flows, respectively. The good agreement between the simulations and experimental measurements for single-phase gas and liquid flows indicates that the new models are reliable and the selected correlations are appropriate.

There are few experimental research results in the open literature for multiphase heat transfer in annuli. The unified Zhang et al. model (2006) is verified by comparison with Manabe’s experimental results for different flow patterns in a crude-oil/natural gas system, and good agreement has been observed in the comparison. So, the unified Zhang et al. model is modified to calculate the heat transfer of gas–liquid flow in annuli based on the “hydraulic diameter” of the annuli, Eq. (17), and the results are compared with the present mechanistic heat-transfer model for gas–liquid flow in annuli.

**Fig. 8:**
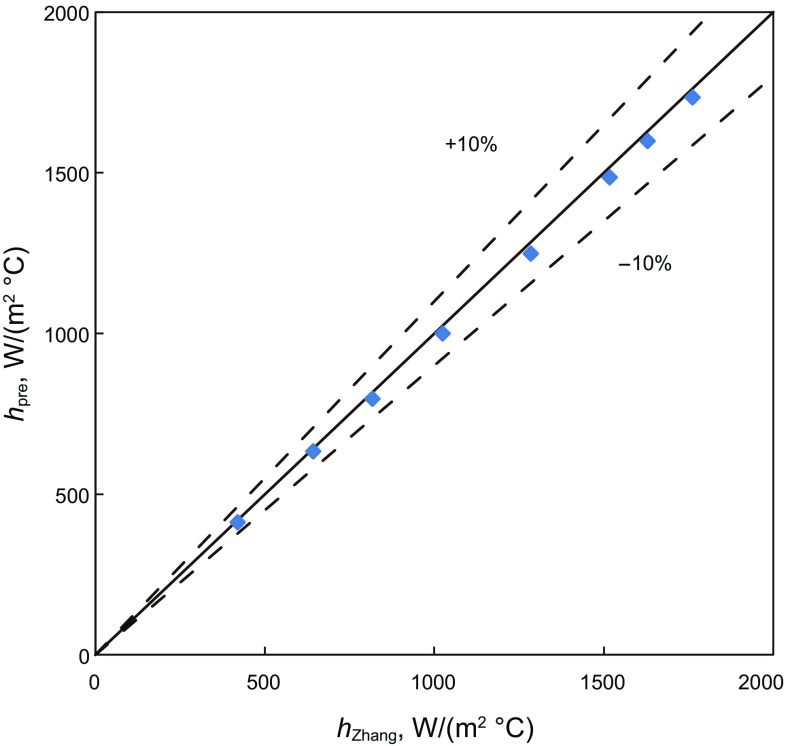
The simulated convective-heat-transfer coefficient comparison between different bubble flow models in vertical annuli

**Fig. 9:**
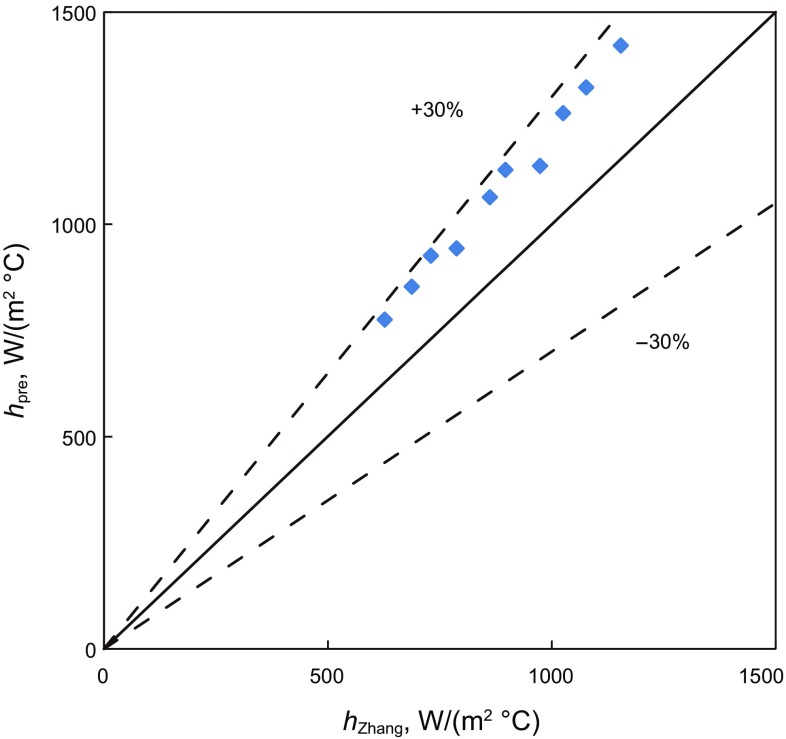
The simulated convective-heat-transfer coefficient comparison between different annular flow models in vertical annuli

**Fig. 10:**
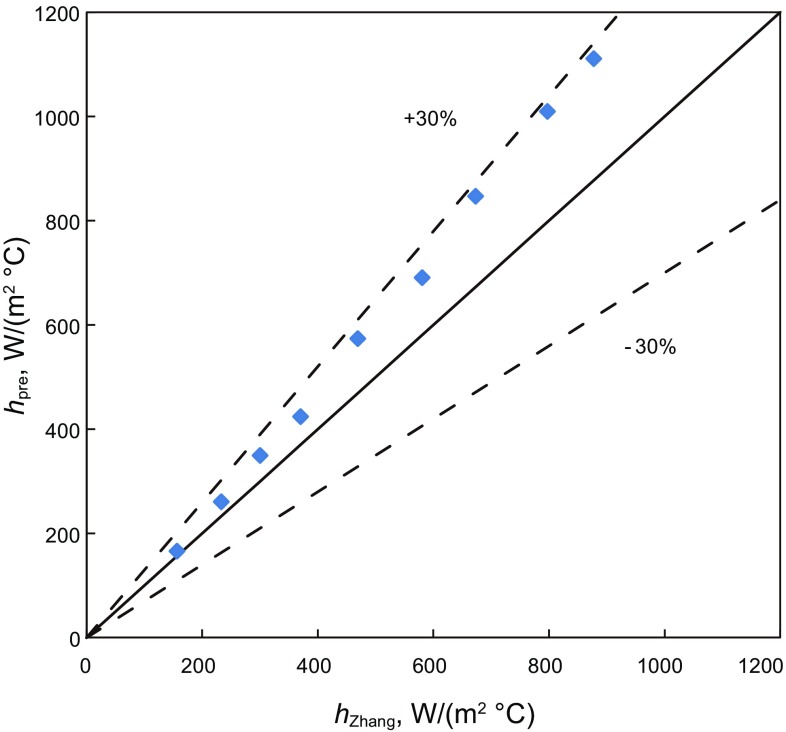
The simulated convective-heat-transfer coefficient comparison between different slug flow models in vertical annuli

Figures 8, 9, and 10 are comparisons of convective-heat-transfer coefficient for bubble flow, annular flow and slug flow predicted by the present model and the modified unified Zhang et al. model (2006). For the bubble flow, the data points are located inside the 10% error band. The agreement is good. It shows that the influence of annulus geometry is small for the low gas volume fraction. For the annular and slug flow in annuli, most of the data points are located inside the 30% error band and all are overestimated. It may because there is a tubing liquid film and a casing liquid film in the wall and the geometry is different from the modified unified Zhang et al. model. It may cause the hydraulic parameters and fluid physical properties to change a lot, leading to larger convective-heat-transfer coefficients.

## Conclusions and discussion

A heat-transfer model for gas–liquid flow in vertical annuli is developed in conjunction with the mechanistic hydrodynamic model of Yin et al. (2014), which can predict flow pattern transitions, liquid holdup, gas void fraction, pressure gradient, and slug characteristics in gas–liquid two-phase flow in vertical annuli. The heat-transfer modeling is based on energy-balance equations and analyses of the temperature differences and variations in the tubing liquid film, casing liquid film, gas core, Taylor bubble and slug body.

The heat-transfer model for single gas or liquid flow is verified by comparison with Manabe’s experimental results (2001). Good agreement has been observed in the comparison. For different flow patterns, it is compared with unified Zhang et al. model modified based on “hydraulic diameter”. For bubble or dispersed-bubble flow, the error is lower than 10%. With the gas void fraction and gas flow velocity increasing, the error will be larger but lower than 30%. In other words, the difference between the new model and modified unified Zhang et al. model will be small if the gas void fraction and velocity is small. The difference will be large when it changes. The modified method based on “hydraulic diameter” is no longer applicable for slug and annular flow in vertical annuli when the gas void fraction increases. It may be 1.3 times larger than the new model. Experimental investigations of heat transfer in vertical are required to improve the model performance.
